# Intrapulmonary and Systemic Pharmacokinetics of Colistin Following Nebulization of Low-Dose Colistimethate Sodium in Patients with Ventilator-Associated Pneumonia Caused by Carbapenem-Resistant *Acinetobacter baumannii*

**DOI:** 10.3390/antibiotics13030258

**Published:** 2024-03-14

**Authors:** Dong-Hwan Lee, Shin-Young Kim, Yong-Kyun Kim, So-Young Jung, Ji-Hoon Jang, Hang-Jea Jang, Jae-Ha Lee

**Affiliations:** 1 Department of Clinical Pharmacology, Hallym University Sacred Heart Hospital, Hallym University College of Medicine, Anyang 14066, Republic of Korea; dhlee@hallym.or.kr; 2 Department of Internal Medicine, St. Vincent’s Hospital, College of Medicine, The Catholic University of Korea, Suwon 16247, Republic of Korea; newzero82@catholic.ac.kr; 3 Division of Infectious Diseases, Department of Internal Medicine, Hallym University Sacred Heart Hospital, Hallym University College of Medicine, Anyang 14066, Republic of Korea; amoureuxyk@hallym.or.kr; 4 Department of Dermatology, Inje University Haeundae Paik Hospital, Busan 48108, Republic of Korea; h00274@paik.ac.kr; 5 Division of Pulmonology and Critical Care Medicine, Inje University Haeundae Paik Hospital, Busan 48108, Republic of Korea; h00596@paik.ac.kr (J.-H.J.); h00075@paik.ac.kr (H.-J.J.)

**Keywords:** colistin, colistimethate sodium, ventilator-associated pneumonia, carbapenem-resistant *Acinetobacter baumannii*, inhalation, nebulization, population pharmacokinetics, epithelial lining fluid, plasma

## Abstract

Colistimethate sodium (CMS) nebulization is associated with reduced systemic toxicity compared to intravenous injection, with potentially enhanced clinical efficacy. This study aimed to assess the pharmacokinetic (PK) properties of colistin during low-dose CMS nebulization in patients with ventilator-associated pneumonia (VAP) caused by carbapenem-resistant *Acinetobacter baumannii*. A nonlinear mixed-effects modeling approach was applied to develop population PK models for colistin in both epithelial lining fluid (ELF) and plasma. Twenty patients participated, and 80 ELF and 100 plasma samples were used for model development. Median colistin concentrations measured in ELF were 614-fold, 408-fold, and 250-fold higher than in plasma at 1, 3, and 5 h, respectively. Time courses in both ELF and plasma were best described by a one-compartment model with a Weibull absorption process. When the final model was simulated, the maximum free concentration and area under the free colistin concentration–time curve at steady state over 24 h in the plasma were approximately 1/90 and 1/50 of the corresponding values in ELF at steady state, respectively. For an *A. baumannii* MIC of 1 mg/L, inhaling 75 mg of CMS at 6 h intervals was deemed appropriate, with dose adjustments needed for MICs exceeding 2 mg/L. Using a nebulizer for CMS resulted in a notably higher exposure of colistin in the ELF than plasma, indicating the potential of nebulization to reduce systemic toxicity while effectively treating VAP.

## 1. Introduction

Ventilator-associated pneumonia (VAP) is commonly characterized as an infectious ailment prevalent among critically ill patients. It typically manifests 48 h or more following endotracheal intubation or the commencement of mechanical ventilation [1]. The mortality rate associated with VAP ranges from 24% to 50%, and VAP significantly increases the economic burden on both patients and society due to prolonged stays in intensive care units and heightened healthcare expenditures [2,3,4,5]. Recent data from the United States regarding the etiological agents of VAP reveal an increasing prevalence of pathogens such as methicillin-resistant *Staphylococcus aureus* (MRSA), multidrug-resistant *Pseudomonas aeruginosa*, and carbapenem-resistant *Acinetobacter baumannii* (CRAB), which are progressively posing challenges, if not failures, to treatment using conventional antibiotics [6,7]. Furthermore, VAP caused by these drug-resistant pathogens is associated with high mortality rates and is increasingly prevalent, posing a globally significant and grave concern [8]. In the Republic of Korea, there has been a steady rise in resistance of Gram-negative bacteria to antibiotics, with a significant increase in carbapenem resistance in *A. baumannii* from 20% in 2002 to 85% in 2015 [9].

Worryingly, there are few effective antibiotics against CRAB. Colistin, which was developed decades ago but was not used due to concerns about toxicity and adverse effects, has now emerged as the sole therapeutic option for CRAB and is increasingly being used [10,11]. Specifically, the use of intravenous (IV) colistin has been a subject of controversy due to its relatively low concentration within the pulmonary parenchyma compared to blood levels and its association with a higher risk of toxic side effects [12]. Consequently, efforts have been made to explore the application of nebulized colistin [13]. Multiple studies have demonstrated that nebulized colistin minimizes systemic side effects and exhibits clinical efficacy in the treatment of VAP. As a result, the Infectious Disease Society of America and the American Thoracic Society, in their 2016 guidelines for VAP management, recommended considering the use of adjunctive colistin inhalation alongside IV injection as part of the treatment regimen for patients with VAP caused by CRAB [14]. The administration of colistin via inhalation has been shown to reduce systemic toxicity compared to IV injection, potentially enhancing its clinical efficacy. However, there is a lack of research on the pharmacokinetics (PK) of colistin in epithelial lining cells and plasma when administered through nebulization.

The objective of this study was to evaluate the pharmacokinetic (PK) properties of colistin by constructing a population PK model in both epithelial lining fluid (ELF) and plasma during low-dose colistimethate sodium (CMS) nebulization in patients with VAP caused by CRAB. Additionally, this research aimed to compare the PK/pharmacodynamic (PD) index obtained through the use of the colistin PK model with the therapeutic efficacy of colistin.

## 2. Results

### 2.1. Study Population

Twenty patients were prospectively enrolled in this study, as shown in Table 1. Primary diseases among the patients were respiratory disease (*n* = 13), cardiovascular disease (*n* = 4), and cancer (*n* = 3). Identified pathogens were *A. baumannii* (*n* = 20), *Pseudomonas aeruginosa* (*n* = 3), *Chryseobacterium indologenes* (*n* = 1), coagulase-negative staphylococci (*n* = 1), *Escherichia coli* (*n* = 1), *Klebsiella pneumoniae* (*n* = 1), and *Stenotrophomonas maltophilia* (*n* = 1).

### 2.2. Colistin A and B Assay in BAL Fluid and Plasma

The lower limits of quantitation for colistin A and colistin B were 3.72 ng/mL and 16.28 ng/mL, respectively. The coefficient of determination was greater than 0.99 for all three batches per day for colistin A and colistin B, indicating the linearity of the calibration curve over the ranges of 3.72–1860 ng/mL and 16.28–8140 ng/mL, respectively. In the daily analysis results, the concentration precision and accuracy of the calibration standard for colistin A were 1.30–12.91% and 92.80–105.15%, respectively, at concentrations of 3.72, 9.3, 18.6, 93, 186, 930, and 1860 ng/mL Those for colistin B were 0.66–12.23% and 86.60–107.92%, respectively, at concentrations of 116.28, 40.7, 81.4, 407, 814, 4070, and 8140 ng/mL. In the inter-day analysis of colistin A, precision was 2.62%, 1.59%, and 5.24% at concentrations of 11.16 ng/mL, 186 ng/mL, and 1488 ng/mL, respectively. Accuracy was 98.65%, 96.65%, and 94.52% at the same concentrations. The inter-day analysis of colistin B revealed precision values of 5.62%, 1.22%, and 5.09% at concentrations of 48.84 ng/mL, 814 ng/mL, and 6512 ng/mL, respectively. Accuracy was 98.45%, 99.79%, and 91.43% at the corresponding concentrations.

### 2.3. Population PK Analysis

**Figure 1:**
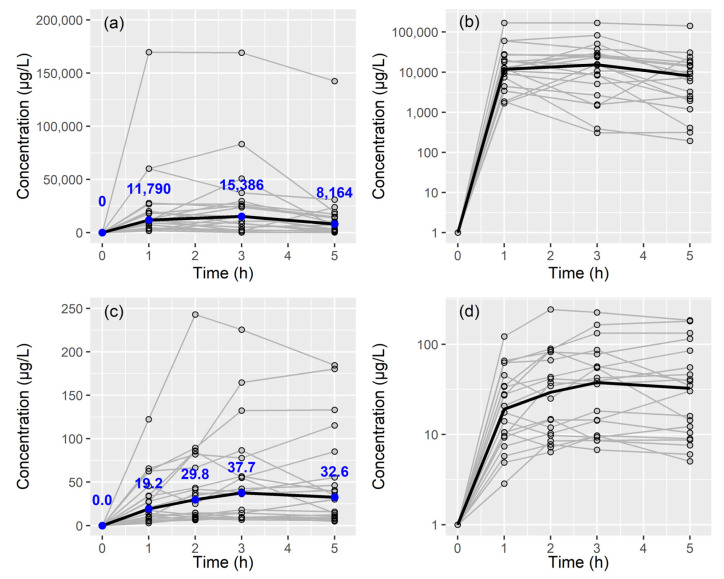
Colistin concentration–time plot of colistin following administration of colistimethate sodium (CMS) 0.9375 MIU for 30 min (equivalent to 75 mg CMS or 28.125 mg colistin-based activity): (**a**) epithelial lining fluid—linear scale; (**b**) epithelial lining fluid—semi-log scale; (**c**) plasma—linear scale; and (**d**) plasma—semi-log scale. Black thick lines, blue dots, and blue numbers represent median values.

Eighty ELF and one hundred plasma samples were used to develop population PK models for colistin. Time-varying PK profiles of colistin in ELF and plasma are shown in Figure 1. Colistin concentrations in ELF measured at 1, 3, and 5 h were approximately 614-fold, 408-fold, and 250-fold higher, respectively, than those in plasma.

**Figure 2:**
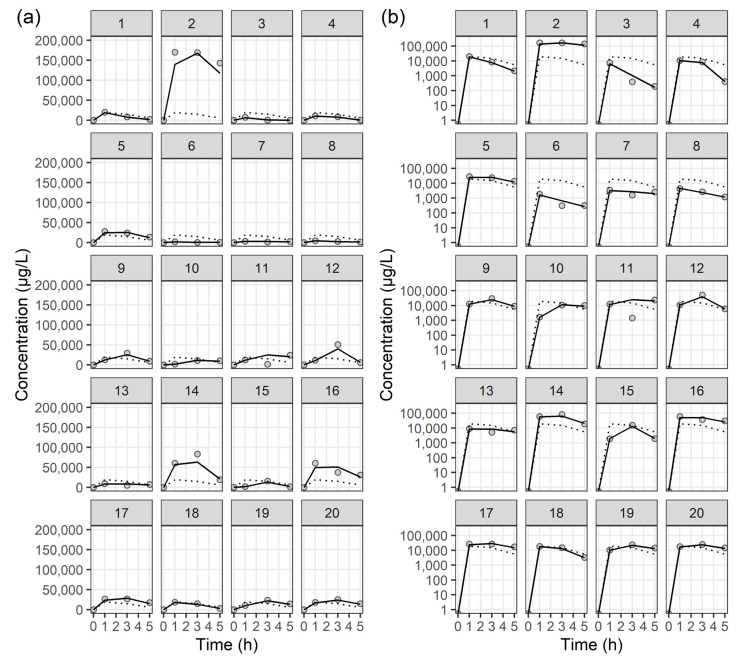
Individual fit plots for the epithelial lining fluid concentration of colistin. (**a**) Normal scale, and (**b**) semi-log scale: observed concentrations (closed circle), individual-predicted concentrations (solid line), and population-predicted concentrations (dotted line).

**Figure 3:**
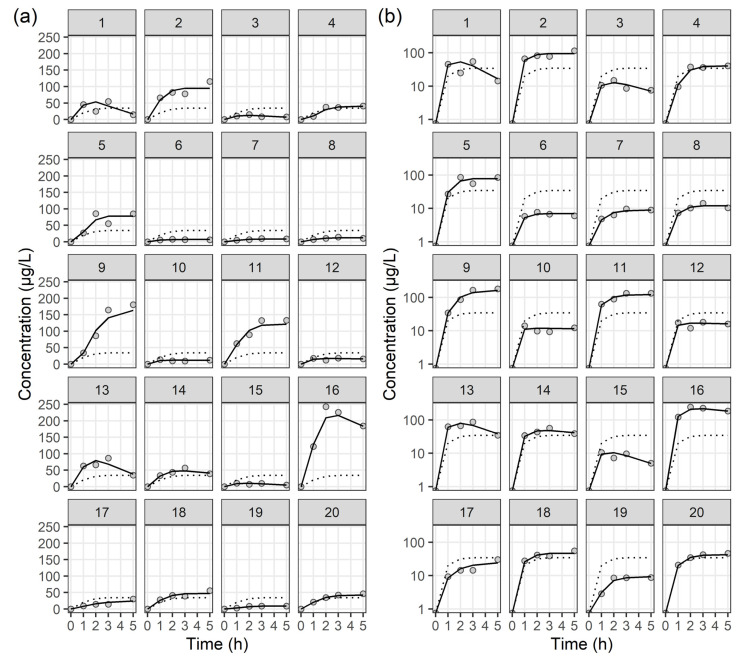
Individual fit plots for the plasma concentration of colistin: (**a**) normal scale, and (**b**) semi-log scale. Closed circles represent observed concentrations, solid lines indicate individual-predicted concentrations, and dotted lines indicate population-predicted concentrations.

The time courses of colistin concentrations in ELF and plasma were best described using one-compartment models with a Weibull absorption process [15]. The following structural PK model described by two differential equations was applied:

(1) $$\frac{\mathrm{dA}1}{\mathrm{dt}}=-\mathrm{WB}\times A1$$

(2) $$\mathrm{WB}=\mathrm{KAMAX}\times \left(1-{e^{\left(-\mathrm{RA}\times \mathrm{time}\right)}}^{\mathrm{GAM}}\right)$$

(3) $$\frac{\mathrm{dA}2}{\mathrm{dt}}=\mathrm{WB}\times A1-\frac{\mathrm{CL}}{V}\times A2$$

where A1 and A2 represent the amounts of colistin in ELF and plasma, and WB denotes the Weibull absorption function with parameters KAMMA, RA, and GAM. KAMMA determines the function’s maximum value, often representing the maximum rate of absorption or event occurrence. RA, the shape parameter, controls the rate of change over time, influencing the distribution’s slope and how quickly it increases or decreases. GAM adjusts the time variable’s exponent, controlling the rate of growth or decline over time, thereby affecting the distribution’s asymmetry and indicating the curve’s speed in reaching its peak or declining. CL and V are the total clearance and volume of distribution for colistin, respectively. The estimated CL and V in Table 2 were expressed as CL/*f*m and V/*f*m, respectively, where *f*m represents the fraction of CMS metabolized to colistin, a value that cannot be estimated from our study data. No significant covariates influencing the PK parameters were identified. The estimated PK parameters for the final PK models are presented in Table 2, while individual fit plots for colistin in ELF and plasma can be found in Figure 2 and Figure 3, respectively. Diagnostic goodness-of-fit plots for the final PK model of colistin in ELF and plasma are shown in Supplementary Materials Figures S1 and S2, respectively. Most CWRES and concentrations were evenly distributed around the *x*-axis or line of equality, indicating that the final structural models were adequate and PK parameters were minimally biased. VPC plots for the final PK model of colistin in ELF and plasma are shown in Figures S3 and S4, respectively. With the observed 10th, 50th, and 90th percentiles mostly within the 95% confidence intervals of the simulated percentiles, the final PK models effectively explained the observed concentrations and had good predictive performance. These results indicate that the final PK models are reliable in the prediction of PK parameters for colistin in ELF and plasma, respectively.

### 2.4. PK/PD Properties

Statistically, there were no significant differences among the groups with respect to area under the free colistin concentration–time curve at steady state over 24 h (*f*AUC), clinical outcomes, or microbiological outcomes (Table 3). When clinical outcomes were compared using mean values, the *f*AUC in patients of the cured/improved group was approximately 27.2 times higher in ELF than in plasma, and that for the failed group was about 14.0 times higher. In ELF, the *f*AUC of colistin was more than 2.25 times higher in cured/improved patients than in those with treatment failure. When clinical outcomes were compared using median values, the *f*AUC in the cured/improved group was approximately 26.2 times higher in ELF than in plasma, and that for the failed group was 15.9 times higher. In ELF, the *f*AUC of colistin was 1.16 times higher in cured/improved patients than in those patients in whom treatment failed.

Microbiologically, when mean results were compared, the *f*AUC in the eradicated group was 39.8 times higher in ELF than in plasma, while that in the persistent group was 18.8 times higher, and that in the indeterminate group was 6 times higher. The eradicated group exhibited an ELF *f*AUC 3.48 times higher than the persistent group and 3.24 times higher than the indeterminate group. Microbiologically, when results were compared using median values, the *f*AUC in the eradicated group was 26.0 times higher in ELF than in plasma, while that in the persistent group was 18.2 times higher, and that in the indeterminate group was 6 times higher. The eradicated group exhibited an ELF *f*AUC 2.15 times higher than the persistent group and 1.80 times higher than the indeterminate group.

**Figure 4:**
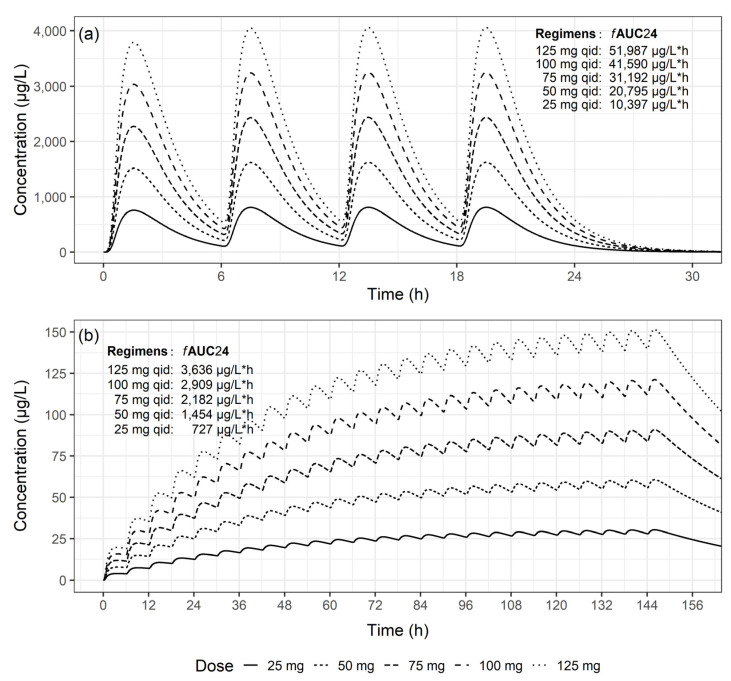
Simulated unbound concentration–time profiles of colistin following nebulization of colistimethate sodium (CMS) at various CMS doses after 30 min in a typical patient: (**a**) epithelial lining fluid, and (**b**) plasma.

Figure 4 shows the concentration–time profiles of unbound colistin simulated using typical parameters from the final PK models constructed from ELF and plasma samples. In the ELF, near-steady-state levels were achieved within 1 day, while that in plasma required 6 days to approach near-steady-state levels. However, even upon reaching steady-state in plasma, both the maximum concentration and *f*AUC values were much lower, by approximately 1/90 and 1/50, respectively, than those observed in the ELF.

## 3. Discussion

In the present study, we quantitatively analyzed the PK properties of colistin following low-dose CMS nebulization in ELF and plasma. Colistin is an effective antibiotic for the treatment of CRAB, a causative pathogen in VAP [16]. However, its nephrotoxicity and neurotoxicity pose challenges when using colistin [17,18]. To address this issue, clinical studies and pharmacokinetic research have been conducted on the clinical efficacy and pharmacokinetics of CMS nebulization, either as adjunctive therapy or as an alternative to IV colistin administration [19,20]. Nevertheless, there is a paucity of population PK research on CMS nebulization, which is why we investigated population PK characteristics in the ELF and plasma.

In our study, patients received a 30 min nebulized dose of 0.9375 million international unit (MIU) of CMS, equal to 75 mg of CMS or 28.125 mg of colistin-based activity (CBA), with concentration monitored over a 5 h period. Our study utilized nebulized CMS at doses below 4 MIU/day, contrary to recent recommendations advocating for higher doses up to 15 MIU/day [13,21]. However, previous retrospective studies have shown similar clinical efficacy at lower doses, especially for susceptible Gram-negative bacilli or *A. baumannii*. A meta-analysis by Vardakas et al. included 11 studies, all employing doses of 4 MIU/day or lower. Despite one study using a higher dosage of 12 MIU/day, the meta-analysis reported a clinical and microbiological success rate of approximately 70%, consistent with our observed efficacy in previous studies using higher CMS doses [22].

The observed median (IQR) peak colistin concentration was 21,950 (10,440–28,389) μg/L in the ELF, while that in plasma was 43.51 (14.23–85.78) μg/L. Boisson et al. reported that, regardless of concentration, a significant adsorption of CMS to the mini-BAL device was not observed in studies utilizing the mini-BAL device. They also noted that non-specific adsorption was not significant at a concentration of 6 mg/L. Consequently, it appears from our study that there was no significant adsorption of CMS [23]. Comparatively, a previous study by Kyriakoudi et al., using 2 MIU of CMS, equivalent to 160 mg of CMS sulfate or 60 mg of CBA, administered via vibrating mesh and jet nebulizers, reported median (IQR) maximum ELF concentrations of 10.4 (4.7–22.6) mg/L and 7.4 (6.2–10.3) mg/L, respectively. Plasma colistin concentrations in that study were documented as 2.6 (2.0–3.5) mg and 0.3 (0.3–1.6) mg for vibrating mesh and jet nebulizers, respectively [20]. Boisson and colleagues administered 2 MIU of CMS via aerosol and reported a wide range of ELF concentrations ranging from 9.53 to 1137 mg/L. Interestingly, the plasma colistin concentrations observed in their study, ranging from 0.15 to 0.73 mg/L, are closely aligned with those measured in our study, emphasizing the consistency in plasma colistin concentrations despite the significant variance in ELF concentrations between studies [24]. These findings collectively indicate that administering CMS via nebulization yields high colistin concentrations in the ELF, markedly higher than those in plasma. This suggests that nebulization as a mode of administration is a favorable method for reducing systemic toxicity, particularly nephrotoxicity.

Our pharmacometric PK modeling results for colistin indicated that an optimal fit was achieved with a one-compartmental model incorporating Weibull absorption and first-order elimination for both epithelial lining fluid and plasma. Although CMS was administered via nebulization at a constant rate for 30 min, the transition of CMS from BALF to ELF and the metabolism of CMS to colistin in either ELF or plasma did not conform to zero-order kinetics. Boisson et al. [24] and Gkoufa et al. [25] developed models encompassing ELF and plasma compartments for nebulized CMS and formed colistin, allowing the bidirectional movement of CMS and colistin between the ELF and plasma. As we did not measure CMS concentrations in this study, the PK processes observed in prior studies were simplified into empirical one-compartment models for colistin. Within these simplified PK processes, we modeled the absorption process using first-order, Weibull, and transit absorption mechanisms and found that the Weibull model provided the best fit. The utilization of the Weibull absorption process in colistin PK modeling has not been employed prior to this study; this absorption process was originally used to model the absorption process post IV administration of isavuconazole [15].

According to our PK model, the estimated V/*f*m of colistin in the ELF was 23 mL, significantly larger than the 1.2 mL reported in the study by Boisson et al. [24] and noticeably different from the 14.5 mL in Gkoufa et al. [25]. These studies measured concentrations of both CMS and colistin in the ELF, assuming equal V/*f*m for CMS and colistin. In our study, the V/*f*m of colistin in plasma was estimated at 67.4 L, which is much greater than the 13.7 L reported in Boisson et al. [24]. Gkoufa et al. fixed this distribution volume at 13.7 L based on Boisson et al. [25]. The CL/*f*m of colistin in the ELF in our model was estimated at 0.541 mL/min. Our simple model, without direct consideration of intercompartment clearance between ELF and plasma, yielded a value similar to Gkoufa et al.’s intercompartmental CL/*f*m of 0.372 mL/min [25]. In our model, the CL/*f*m of colistin in plasma was 26.3 mL/min, notably lower than the 53.1 mL/min in Boisson et al. [24] and the 128 mL/min in Gkoufa et al. [25]. This discrepancy could be due to the shorter dosing intervals in our study, leading to limited information on colistin elimination from blood samples due to the extended absorption of colistin.

We investigated whether the *f*AUC values of individual patients were associated with clinical or microbiological outcomes (Table 3). Using individual estimates of CL derived from the final PK model, we calculated the steady-state *f*AUC for each patient when a total of 3.75 MIU (=300 mg CMS = 112.5 mg CBA) was administered at 6 h intervals four times a day, using the formula AUC = DOSE/CL. The unbound fraction of colistin was assumed to be 10% [25] in the ELF based on the study by Gkoufa et al. and 34% in the plasma based on the study by Mohamed et al. [26]. Gkoufa et al. presented concentration–time profiles of colistin predicted through simulations, assuming unbound fractions in the ELF of 0.01, 0.015, 0.05, and 0.099 [25]. From these, we approximated the unbound fraction at 0.1 by rounding 0.099. Meanwhile, Mohamed et al. determined the unbound fraction in plasma using equilibrium dialysis and reported a measured median (range) colistin unbound fraction of 34% (26–41%) [26]. In our study, the mean (SD) *f*AUC/MIC in the ELF was 63.2 (109) mg/L·h, with a median (range) of 36.7 (1.92–507) mg/L·h. While no statistically significant difference in microbiological outcomes was observed, eradicated patients tended to exhibit higher *f*AUC values than persistent or indeterminate patients. The *f*AUC/MIC target values for a 2 log10 kill of *A. baumannii* by colistin were 17.5–43.0 mg/L·h and 22.5–95.0 mg/L·h for murine thigh and lung infection models, respectively [27]. Assuming the MIC to be 1 mg/L, among our study patients, seven had values below 22.5 mg/L·h. Of these, two clinically failed, and four did not achieve microbiological eradication. Among the 13 patients with values above 22.5 mg/L·h, 11 clinically succeeded, while 8 did not achieve microbiological eradication. According to MIC distributions reported by the European Committee on Antimicrobial Susceptibility Testing, among 2879 isolates, 805 (30.0%) had a colistin MIC of 0.5 mg/L, 1451 (50.4%) had an MIC of 1 mg/L, 539 (18.7%) had an MIC of 2 mg/L, and 54 (1.88%) had an MIC of 4 mg/L. In cases with MICs equal to or exceeding 2 mg/L, dose escalation may be warranted [28].

Our study had several limitations. First, we utilized nebulized CMS at doses below 4 MIU/day, contrary to recent recommendations advocating for higher doses up to 15 MIU/day [13,21]. However, previous retrospective studies have shown similar clinical efficacy at lower doses, especially for susceptible Gram-negative bacilli or *A. baumannii*. A meta-analysis by Vardakas et al. included 11 studies, all employing doses of 4 MIU/day or lower. Despite one study using a higher dosage of 12 MIU/day, the meta-analysis re-ported a clinical and microbiological success rate of approximately 70%, consistent with our observed efficacy in previous studies using higher CMS doses [22]. Second, despite the predominance of VAP occurrence in the lower lobes, we sampled from the right middle lobe and lingula. Consequently, there is a potential for an overestimation of ELF concentrations, given the likelihood of aerosolized particles predominantly reaching aerated lung regions. However, conducting the BAL procedure uniformly in the right middle lobe over a short period was necessary to ensure consistency and reliability in our results. Third, nebulization parameters (such as the type and position of nebulizers, ventilator settings, and coordination with the ventilator) were not optimized. This was due to the diverse and often severe condition of registered VAP patients, making it ethically and medically challenging to arbitrarily adjust the settings and modes of mechanical ventilators according to the research objectives. Fourth, we were unable to measure the concentration of CMS, resulting in the development of a simplified PK model and failure to establish a model linking ELF and plasma. However, a complex model is not always superior, and our internal validation demonstrated that our model effectively described the observed data. Fifth, the significant deposition of aerosolized particles on bronchial walls results in bronchial pharmacokinetics that may not accurately reflect interstitial lung tissue concentrations [29]. Consequently, we adopted a bioavailability assumption of 9% from the literature to calculate the PK/PD index. Sixth, it has been noted that there is considerable variability in the observed colistin levels in our study. There were more than 100-fold differences between the minimum and maximum levels. This suggests that the variability may be attributed to sampling issues or technical variability. It is suggested that future research should consider better sampling techniques and more consistent technical approaches. Seventh, we could not identify covariates influencing PK parameters, likely due to the limited number of patients. Eighth, we did not find a significant correlation between *f*AUC and treatment outcomes. Future research should investigate MIC values to calculate *f*AUC/MIC and to enhance our understanding of the relationship between the therapeutic effects of colistin on *A. baumannii* and its PK/PD index.

In conclusion, when CMS was administered via a nebulizer, the measured colistin concentrations in the ELF were 250–614 times higher than those in plasma. Therefore, administering CMS via nebulization, without concomitant intravenous administration, is believed to contribute to reducing systemic toxicity when treating VAP. Colistin PK following CMS nebulization was well described by a one-compartment PK model with Weibull absorption and linear elimination in both ELF and plasma. When the MIC of *A. baumannii* was 1 mg/L, inhaling 75 mg of CMS at 6 h intervals was appropriate, but doses needed to be increased for cases exceeding 2 mg/L. Due to the current dearth of research on the PK/PD indices of colistin and its treatment efficacy against CRAB, further studies are needed to determine appropriate colistin nebulization dosage regimens that show good efficacy but minimal systemic toxicity.

## 4. Materials and Methods

### 4.1. Study Population

The Institutional Review Board of Inje University Haeundae Paik Hospital (IRB No. 2020-06-019) approved this study, which adhered to the principles of the Declaration of Helsinki and Good Clinical Practice. Prior to inclusion in the study, written informed consent forms were signed by the legal representatives of all unconscious patients. The research focused on adult patients hospitalized in the intensive care unit (ICU) of Haeundae Paik Hospital in Busan, Republic of Korea, who developed ventilator-associated pneumonia during their admission between August 2020 and December 2022.

Patients eligible for participation in the clinical study met the following criteria:

- Adults aged 18 years and older, who had undergone endotracheal intubation or initiation of mechanical ventilation, and were diagnosed with VAP.
- Pneumonia was defined as the presence of new or worsening radiographic opacities on chest X-rays, accompanied by two or more of the following criteria: fever exceeding 38 °C, purulent tracheal secretions, and an elevated (>11,000/µL) or reduced white blood cell count (<4000/µL).
- VAP was defined according to the 2016 clinical practice guideline by the Infectious Diseases Society of America and the American Thoracic Society [14] as pneumonia in patients receiving mechanical ventilation that occurred at least 48 h after endotracheal intubation.
- Detection of CRAB bacteria exclusively in respiratory samples obtained through BAL or endobronchial aspiration, based on the specified criteria (growth thresholds considered significant at 10^3^ colony forming unit (CFU)/mL for endotracheal bronchial aspiration and 10^4^ CFU/mL for BAL fluid).
- The quality and suitability of the samples was judged according to Murray and Washington’s grading system [30], and only samples belonging to Grade 5 (epithelial cell count < 10/LPF, white blood cell count > 25/LPF).
- Patients were not eligible to participate if their legal representative did not provide consent or if the infecting pathogen was not susceptible to colistin.

### 4.2. Colistin Administration and Sampling Procedures

Patients were treated with CMS (Xellia Pharmaceuticals ApS, Copenhagen, Denmark) at a total of 3.75 MIU (equivalent to 300 mg of CMS or 112.5 mg of CBA) administered four times a day at 6 h intervals. CMS was dissolved in 5 mL of normal saline and administered over 30 min through a jet nebulizer (Sileo 54, Macjin Medical, Seoul, Republic of Korea) connected to the ventilator circuit. Bronchoalveolar lavage fluid (BALF) was obtained by instilling 20 mL of normal saline into the right middle lobe or left lingular segmental bronchus using a bronchoscope, followed by immediate aspiration and collection into a syringe. Samples were collected immediately before the nebulization treatment and at 1, 3, and 5 h after the start of the treatment. Blood samples were collected before the nebulization treatment and at 1, 2, 3, and 5 h thereafter. Both BALF and blood samples were centrifuged at 3000× *g* for 10 min within 1 h of collection and the supernatant was isolated. Samples were then stored in a refrigerator at a temperature below −70 °C.

### 4.3. Colistin Assay in BAL Fluid and Plasma

A validated liquid chromatography–tandem mass spectrometry assay was used to determine colistin concentrations in BALF and plasma samples. The HPLC equipment consisted of an Agilent 1200 series modular system and an Atlantis C18 column (Company, Waters, Milford, MA, USA) (2.1 mm × 150 mm, 3.0 μm). Mobile phases A and B were water and acetonitrile, respectively. Both phases contained 0.1% formic acid. A gradient elution program was started at 10% B, increased to 35% for 1 min, maintained at this composition for 3.8 min, and returned to the initial composition for 5 min to re-equilibrate the column. Mass spectrometry (MS) was performed using a triple-quadruple mass spectrometer (SCIEX QTRAP5500, Applied Biosystems, Foster City, CA, USA) with an electrospray ionization (ESI) interface. The ESI source was set to positive ionization mode. The parent to product transition (m/z) was 578.483→101.071 for colistin A, 585.453→101.071 for colistin B, and 602.527→101.000 for polymyxin B. Data acquisition and processing were performed using the Analyst software (version 1.7.1; Applied Biosystems, Foster City, CA, USA). Stock solutions of colistin and IS were prepared in deionized water at concentrations of 10 mg/mL, and both were stored at −20 °C. Working solutions of colistin were prepared in deionized water (dilutions of 2, 5, 10, 50, 100, 500, and 1000 μg/mL). Calibration standards were prepared by spiking 247.5 μL of drug-free plasma with 2.5 μL of colistin working solution. Low (colistin A; 11.16 ng/mL, colistin B; 48.84 ng/mL), moderate (colistin A; 186 ng/mL, colistin B; 814 ng/mL), and high (colistin A; 1488 ng/mL, colistin B; 6512 ng/mL) concentrations of QC samples were prepared. Sample pretreatment involved a solid phase extraction. A 250 μL aliquot of plasma was pipetted into a 1.5 mL Eppendorf tube, to which 10 μL of IS (10 μg/mL, polymyxin B) and 740 μL of deionized water were added. This mixture was loaded into a cartridge that had been preconditioned twice with 1 mL of methanol and 1 mL of deionized water. After the plasma mixture was loaded, the cartridge was washed with 1 mL of deionized water. One milliliter of methanol including 0.1% formic acid was used for extraction. Extraction liquid was evaporated by vacuum concentrator (Speedvac, Thermo Fisher Scientific Inc., Waltham, MA, USA) at 45 °C for 2.5 h. Samples were reconstituted in 100 μL deionized water containing 0.1% formic acid. Subsequently, samples were vortexed for 5 min and centrifuged at 13,200 rpm for 10 min, and 2 mL of supernatant was injected into the LC-MS system.

Colistin concentrations, encompassing both colistin A and colistin B, were determined and collectively referred to as “colistin”. The concentration of colistin in the epithelial lining fluid (ELF), termed C_ELF_, was calculated using the formula C_ELF_ = C_BAL_ (Urea_plasma_/Urea_BAL_), where C_BAL_ is the colistin concentration measured in BALF. Urea_BAL_ and Urea_plasma_ represents urea concentrations measured in BALF and plasma.

### 4.4. Population PK Analysis

A population PK analysis of colistin in ELF and plasma was conducted using NONMEM 7.5.0 (ICON Clinical Research LLC, North Wales, PA, USA). The analysis used first-order conditional estimation with interaction (FOCEI) to estimate both observed fixed and unexplained random effect parameters. The FOCEI allows the inter-individual variability (IIV) in the PK parameters to interact with the residual unexplained variability (RUV) in the observed concentrations. The RUV arises from factors such as within-subject variability, assay error, and potential misspecification of the model. ADVAN1 TRANS2 and ADVAN3 TRANS4 from the NONMEM PK model library were used to construct the single- and two-compartment models, respectively. Except for drug inhalation, all PK processes were assumed to follow first-order kinetics. Various absorption models, including zero-order, first-order, Weibull, and transit absorption models, were investigated to describe the absorption processes of colistin.

The representation of each PK parameter was given by θi = θ × exp(ηi), where θ stands for the typical value of the PK parameter, θi represents an individual PK parameter, and ηi is a random variable associated with IIV. It was assumed that IIV follows a normal distribution with a mean of 0 and a variance of ω^2^. For RUV, which was assumed to be normally distributed with a mean of 0 and a variance of σ^2^; additive, proportional, and combined additive and proportional error models were investigated.

Models were assessed and chosen based on NONMEM objective function values (OFVs), the precision of parameter estimates (relative standard errors), the shrinkage of IIV, and diagnostic goodness-of-fit plots. In the log-likelihood ratio test, if the decrease in OFV (ΔOFV) is greater than 3.84 for models with one additional degree of freedom or greater than 5.99 for models with two additional degrees of freedom, it is considered statistically significant at *p* < 0.05, indicating an improvement in the model. Diagnostic plots included the following: conditional weighted residuals (CWRES) vs. time, CWRES vs. population predictions (PRED), observations vs. PRED, and observations vs. individual predictions. To identify covariates, models were evaluated with a visual predictive check, and nonparametric bootstrapping was performed. Perl-speaks-NONMEM software (version 5.3.1, [https://uupharmacometrics.github.io/PsN], accessed on 30 April 2023) was used.

Significant covariates for PK parameters were identified through stepwise forward selection and backward elimination. Selection criteria for statistical significance were set at *p* < 0.01 (ΔOFV < −6.635 for one degree of freedom), and elimination criteria were defined as *p* < 0.001 (ΔOFV > 10.83 for one degree of freedom). A covariate was considered significant if it demonstrated both statistical and clinical relevance. Several covariates were examined to assess their impact on the parameters of the population pharmacokinetic model. Demographic factors included in the investigation were sex, age, weight, height, and body surface area. Patient-specific factors included the presence of primary diseases, comorbidities such as hypertension or diabetes, ECMO use, and CRRT use. Laboratory test parameters used in the exploration included serum protein levels, serum albumin levels, serum creatinine levels, serum procalcitonin levels, and arterial blood gas analysis findings. External factors considered during the exploration included the length of hospital stay, length of ICU stay, and length of mechanical ventilation. The influence of renal clearance, estimated using formulae of Cockcroft–Gault [31], Modification of Diet in Renal Disease [32], and Chronic Kidney Disease Epidemiology Collaboration [33], on colistin clearance was also investigated.

Visual predictive checks (VPCs) were conducted using Perl-speaks-NONMEM software (version 5.3.1, [https://uupharmacometrics.github.io/PsN], accessed on 30 April 2023). These checks involved comparing the observed concentrations with the 80% prediction interval derived from 1000 simulated datasets generated from the final PK model. Median and 95% confidence intervals of the PK parameter estimates from 2000 bootstrap samples were calculated to assess potential variability in the predictions of the final PK model. R software (version 4.3.1, [www.rproject.og], accessed on 30 April 2023) was used to post-process the modeling results and for visualization.

### 4.5. PK/PD Properties

The PK/PD index for colistin is the area under the free colistin concentration-–time curve at steady state over 24 h divided by the pathogen minimum inhibitory concentration (*f*AUC/MIC) [27]. Since we were unable to obtain MIC values for *A. baumannii*, we evaluated the relationship between the PK and PD of colistin by comparing the *f*AUC of each patient in accordance with their clinical outcomes and microbiological outcomes. AUC was calculated by dividing the daily dose by patient CL, which was the individual post hoc Bayesian estimate of the developed PK model. The bioavailability of colistin was assumed to be 9% [24], with the assumption of an unbound fraction of 10% in ELF and 34% in plasma [25,26]. The Wilcoxon rank-sum test was used to assess the relationships between *f*AUC and clinical outcomes, and the Kruskal–Wallis test was used to investigate the association between *f*AUC and microbiological outcomes. Additionally, leveraging typical PK parameter values from final models of ELF and plasma samples, we simulated concentration-time profiles of colistin for nebulization doses of 25 mg, 50 mg, 75 mg, 100 mg, and 125 mg in patients. Subsequently, we generated concentration–time plots and computed *f*AUC values.

### 4.6. Definition of Clinical Course Assessment

Clinical outcomes were classified as cured (resolution of symptoms/signs of pneumonia plus improvement or lack of progression in radiologic pulmonary infiltrates, and antibiotics-free), improved (significant improvement of symptoms/signs of pneumonia plus improvement or lack of progression in radiologic abnormalities, but remaining on antibiotic treatment), and failed (persistent or worsening of symptoms/signs of pneumonia, persistent or progression of radiologic pulmonary infiltrates that required additional antibiotic therapy, or death) [34].

## Figures and Tables

**Table 1:** Patient characteristics.

Characteristics	Mean (SD) ^†^ or No. ^‡^ or Median (IQR) ^§^
Demographic characteristics	
Sex	Male 13/Female 7 ^‡^
Age, years	68.9 (12.6) ^†^
Weight, kg	64.9 (15.6) ^†^
Height, cm	167.3 (7.35) ^†^
Clinical characteristics	
Hypertension	Yes 13/No 7 ^‡^
Diabetes	Yes 6/No 14 ^‡^
ICU duration, days	16.5 (12.5–23) ^§^
MV duration, days	14 (11.75–19.25) ^§^
Admission duration, days	44 (32.5–88) ^§^
CMS nebulization duration, days	16 (14–20.25)
ECMO	Yes 4/No 16 ^‡^
CRRT	Yes 2/No 18 ^‡^
Shock	Yes 3/No 17 ^‡^
Clinical outcomes	Cured 16/Failed 4 ^‡^
Microbiologic outcomes	Eradication 8/Persistence 10/Indeterminate 2 ^‡^
Survival	Yes 5/No 15 ^‡^
Laboratory characteristics	
C-reactive protein, mg/dL	8.69 (5.52) ^†^
Albumin, mg/dL	2.75 (0.333) ^†^
Procalcitonin, ng/dL	0.31 (0.2125–1.585) ^§^
Platelets, no.	205 (111) ^†^
Creatinine, mg/dL	0.635 (0.405–1.115) ^§^
ABGA lactate, mmol/L	1.5 (1.15–2.575) ^§^

ICU, intensive care unit; MV, mechanical ventilation; CMS, colistimethate sodium; ECMO, extracorporeal membrane oxygenator; CRRT, continuous renal replacement therapy; ABGA, arterial blood gas analysis; ^†^, mean and standard deviation; ^‡^, number; ^§^, median and interquartile range.

**Table 2:** Parameter estimates and bootstrap medians (95% confidence intervals) for the final PK models of colistin in epithelial lining fluid and plasma.

Parameter	Epithelial Lining Fluid			Plasma		
	Estimates	RSE	Bootstrap Median	Estimates	RSE	Bootstrap Median
		(%)	(95% CI)		(%)	(95% CI)
Structural model						
CL/*f*m (mL/min)	0.541	23.2	0.529 (0.352–0.77)	26.3	27.3	26.1 (6.66–67)
V/*f*m (mL or L)	23.0	28.9	24.3 (11.0–46.7)	67.4	22.2	67.2 (46.6–93)
KAMAX	0.531	16.1	0.533 (0.335–0.973)	1.20	17.2	1.20 (0.912–1.59)
RA	2.16 *			2.70 *		
GAM	2.92 *			1.79 *		
Interindividual variability						
CL/*f*m (%)	124	18.6	124 (96.3–156)	196	16.2	190 (134–303)
V/*f*m (%)	59.7	25.8	58.2 (0.000–106)	111	9.8	110 (94.6–124)
KAMAX (%)	84.3	15.4	84.9 (37.1–109)	64.3	23.3	62.8 (0.0734–86.5)
RA	83.0 *			83.0 *		
GAM	0 *			0 *		
Residual variability						
Proportional error (%)	27.1	18.6	26.7 (19.0–33.3)	18.5	11.3	18.4 (15.8–21.2)

RSE, relative standard error; CI, confidence interval; CL, clearance; V, volume of distribution (mL for epithelial lining fluid, L for plasma); *f*m, fraction of CMS metabolized to colistin; KAMAX, the maximum rate of absorption; RA, shape parameter; GAM. parameter controlling the curve’s growth or decline speed; Weibull absorption rate = KAMAX × (1 − exp(−RA × time)^GAM^); *, fixed value.

**Table 3:** Comparison of clinical and microbiological outcomes based on *f*AUC (mg/L·h) with a total of 3.75 MIU of colistimethate sodium administered at 6 h intervals four times a day.

Outcome	No.	Epithelial Lining Fluid	Plasma
		Median (IQR)	Median (IQR)
Clinical outcomes			
Cured/Improved	16	36.7 (18.2–62.9)	1.40 (0.752–3.74)
Failed	4	31.7 (15.4–47.8)	2.00 (1.87–2.38)
		*p*-value = 0.6366 ^‖^	*p*-value = 0.7055 ^‖^
Microbiological outcomes			
Eradicated	8	61.2 (12.5–111)	2.36 (0.645–4.11)
Persistent	10	28.5 (17.7–50.5)	1.56 (1.00–2.05)
Indeterminate	2	33.9 (30.0–37.9)	5.96 (5.00–6.91)
		*p*-value = 0.5977 ^¶^	*p*-value = 0.1163 ^¶^

*f*AUC, area under the free colistin concentration–time curve at steady state over 24 h; IQR, interquartile range; ^‖^ Wilcoxon Rank-sum test; ^¶^ Kruskal–Wallis test.

## Data Availability

The datasets generated and/or analyzed during the current study are available from the corresponding author on reasonable request.

## Supplementary Materials

The following supporting information can be downloaded at: https://www.mdpi.com/article/10.3390/antibiotics13030258/s1, Figure S1: Goodness-of-fit plots for the final PK models of colistin in epithelial lining fluid: (a) conditional weighted residuals versus time, (b) conditional weighted residuals versus population-predicted concentration, (c) observed concentration versus population-predicted concentration, and (d) observed concentration versus individual-predicted concentration. Figure S2: Goodness-of-fit plots for the final PK models of colistin in plasma: (a) conditional weighted residuals versus time, (b) conditional weighted residuals versus population-predicted concentration, (c) observed concentration versus population-predicted concentration, and (d) observed concentration versus individual-predicted concentration. Figure S3: Visual predictive check for the final PK models of colistin in epithelial lining fluid. Figure S4: Visual predictive check for the final PK models of colistin in plasma.
