# Supplementary material for: Intrapulmonary and Systemic Pharmacokinetics of Colistin Following Nebulization of Low-Dose Colistimethate Sodium in Patients with Ventilator-Associated Pneumonia Caused by Carbapenem-Resistant Acinetobacter baumannii
Source: Antibiotics (Basel). 2024 Mar 14;13(3):258. doi: 10.3390/antibiotics13030258 (PMC10967270; doi:10.3390/antibiotics13030258)
Supplement: Supplementary file 1 [file antibiotics-13-00258-s001.zip › antibiotics-2850732-supplementary.pdf]

Supplementary Materials

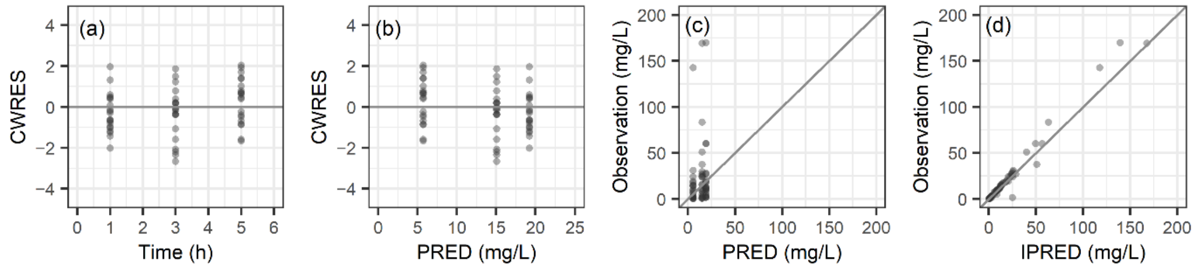

Figure S1. Goodness-of-fit plots for the final PK models of colistin in epithelial lining fluid: (**a**) conditional weighted residuals (CWRES) versus time, (**b**) CWRES versus population predicted concentration (PRED), (**c**) observed concentration versus PRED, and (**d**) observed concentration versus individual predicted concentration (IPRED).

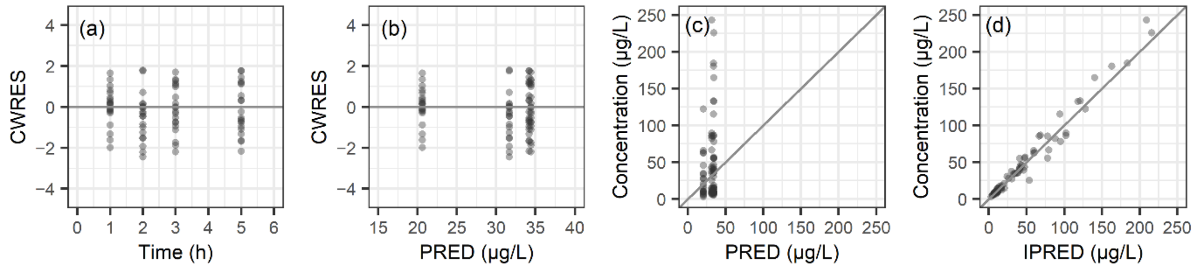

Figure S2. Goodness-of-fit plots for the final PK models of colistin in plasma: (**a**) conditional weighted residuals (CWRES) versus time, (**b**) CWRES versus population predicted concentration (PRED), (**c**) observed concentration versus PRED, and (**d**) observed concentration versus individual predicted concentration (IPRED).

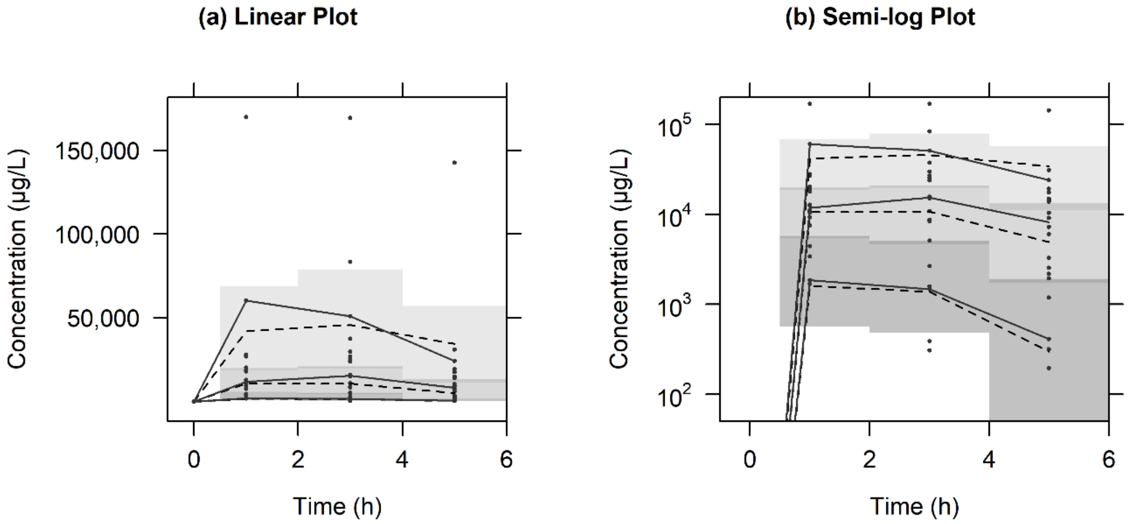

Figure S3. Visual predictive check for the final PK models of colistin in epithelial lining fluid, (**a**) linear plot (**b**) semi-log plot: closed circles, observed concentrations; solid lines, 10th, 50th, and 90th percentiles of observations; dashed lines, 10th, 50th, and 90th percentiles of simulated concentrations; shaded areas, 95% confidence intervals for the 10th, 50th, and 90th percentiles of simulated concentrations.

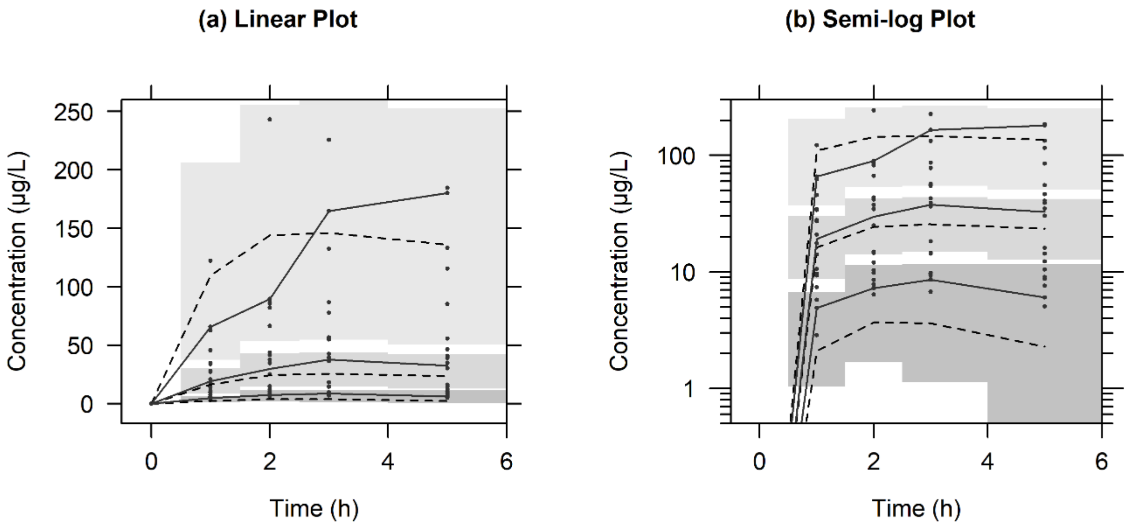

Figure S4. Visual predictive check for the final PK models of colistin in plasma, (**a**) linear plot (**b**) semi-log plot: closed circles, observed concentrations; solid lines, 10th, 50th, and 90th percentiles of observations; dashed lines, 10th, 50th, and 90th percentiles of simulated concentrations; shaded areas, 95% confidence intervals for the 10th, 50th, and 90th percentiles of simulated concentrations.
